# Clinical Features and Prognosis of Acute Cholangitis in Octogenarians: A Prospective Comparative Study

**DOI:** 10.3390/medicina60111759

**Published:** 2024-10-26

**Authors:** Mustafa Comoglu, Fatih Acehan, Enes Seyda Sahiner, Cagdas Kalkan, Ezgi Comoglu, Yusufcan Yılmaz, Tolga Canlı, Ihsan Ates

**Affiliations:** 1 Department of Internal Medicine, Ankara Bilkent City Hospital, Ankara 06800, Turkey; 2 Department of Gastroenterology, Ankara Bilkent City Hospital, Ankara 06800, Turkey; 3 Department of Internal Medicine, Ankara Etlik City Hospital, Ankara 06170, Turkey

**Keywords:** acute cholangitis, biliary drainage, elderly patients, octogenarians, in-hospital mortality

## Abstract

*Background*: The data on acute cholangitis in the octogenarian population are very limited. This study aimed to examine the clinical characteristics of acute cholangitis and complications related to endoscopic retrograde cholangiopancreatography (ERCP) in octogenarians. *Materials and Methods*: This study was conducted prospectively between July 2022 and December 2023 and included 250 patients aged 65 years and older. Patients eligible for the study were divided into two groups: those aged ≥80 years (octogenarian) and those aged 65–79 years (non-octogenarian). These two groups were compared in terms of the clinical characteristics of cholangitis and ERCP-related complications. In addition, factors associated with in-hospital mortality were evaluated. *Results*: Out of 250 patients, 87 (34.8%) were octogenarians and 163 (65.2%) were non-octogenarians. The median age was 76 (69–82) years. Although the octogenarian group had higher rates of severe illness and intensive care unit admissions (*p* < 0.001 and *p* = 0.002, respectively), there were no significant differences in in-hospital mortality (*p* = 0.359) or ERCP-related complications (*p* = 0.417). Malignant etiology (odds ratio [OR]: 5.68, 95% confidence interval [CI]: 2.11–15.3), hypoalbuminemia (OR 0.18, 95% CI 0.07–0.45), and qSOFA score ≥ 2 (OR: 6.5, 95% CI: 1.7–24.5) were independent risk factors for in-hospital mortality. *Conclusions*: Being over 80 years old is not an indicator of poor outcomes, and ERCP can be safely performed on these patients. However, elderly patients with hypoalbuminemia, malignant etiology, or a qSOFA score of ≥2 should be closely monitored, regardless of their age.

## 1. Introduction

Acute cholangitis (AC) is a clinical condition that occurs as a result of infection and is accompanied by impaired bile drainage in the biliary tract. In the etiology of AC, biliary stones and malignancies are the most common causes [1]. Although disease mortality in AC exceeded 20–30% in the past, it has decreased to 2–3% with the development of imaging methods and new treatment modalities [2].

In elderly patients, biliary tract pathologies, such as gallstones and malignancies, are more commonly observed [3]. The global increase in life expectancy also contributes to an increase in the number of patients with biliary tract pathologies [4]. Aging is associated with an increased incidence of cardiovascular diseases, a decrease in vital capacity, and the development of frailty and sarcopenia [5]. In elderly patients, the presence of multiple comorbidities, a decline in functional capacity, and potential delays in diagnosis due to atypical presentations can lead to poor clinical outcomes [6,7]. Additionally, due to the potential complications of interventional procedures such as endoscopic retrograde cholangiopancreatography (ERCP), including pancreatitis and bleeding, performing these procedures in elderly patients may contribute to poor outcomes [8]. In addition to studies showing increased complications in the elderly, conflicting data suggesting no increases have made interventional procedures in this group a concern for clinicians [8,9,10,11,12].

In summary, the existing literature presents conflicting data regarding the clinical course of cholangitis and ERCP complications in elderly patients [12,13,14,15,16]. Furthermore, there is a lack of large-scale prospective studies addressing this issue. A clearer identification of poor prognostic factors in elderly patients will provide clinicians with valuable guidance in terms of decision-making regarding patient management and interventional procedures. Therefore, the present study prospectively investigates the prognosis of AC and ERCP-related complications in both octogenarian and non-octogenarian patients to address these gaps.

## 2. Materials and Methods

This study was conducted prospectively at the Department of Internal Medicine of Ankara Bilkent City Hospital with the approval of the ethics committee (approval number: E1-22-2774). Written, informed consent was obtained from all participants.

### 2.1. Study Design and Clinical Outcomes

The study was conducted between July 2022 and December 2023. Patients referred from the emergency department to the internal medicine or gastroenterology departments for consultation due to suspected AC were evaluated. The inclusion criteria were being 65 years of age or older and meeting the definitive diagnostic criteria for AC according to the Tokyo 2018 Guidelines (TG18) [17]. The exclusion criteria included having a suspected diagnosis of AC that did not meet all three TG18 diagnostic criteria, being under 65 years of age, and not providing consent to participate in the study. Patients eligible for the study were divided into two groups: those aged 80 years and over (octogenarian) and those aged 65–79 years (non-octogenarian). These two groups were compared in terms of clinical and demographic characteristics, laboratory parameters, prolonged hospitalization, mortality, intensive care unit (ICU) admission, and procedural complications. In addition, factors associated with in-hospital mortality were examined and independent risk factors were identified.

Patients were followed-up from admission to discharge. The follow-up and treatment of the patients included in the study were performed in a standardized manner as recommended by current guidelines. Intravenous fluid therapy and appropriate antibiotherapy were started immediately in every patient diagnosed with AC. Biliary drainage was performed according to the severity of the disease, as specified in the TG18 [17,18]. ERCP was usually performed as biliary drainage, while percutaneous drainage was undertaken in cases in which ERCP failed or could not be performed due to technical reasons.

### 2.2. Definitions and Data Collection

The diagnosis and severity classification of AC were made based on the TG18 [17]. All three of the following criteria had to be met for the diagnosis of AC: evidence of systemic inflammation, laboratory evidence of cholestasis, and evidence of biliary dilation or underlying etiology on imaging. Using the Tokyo severity grading (TSG) criteria, the patients were divided into three categories: mild AC (grade 1), moderate AC (grade 2), and severe AC (grade 3). Prolonged hospitalization was defined as a hospital stay of >17 days, since 17 days represented the value above the 75th percentile of the hospital stay of the entire study population. General demographic characteristics, presenting complaints, vital signs, quick sequential organ failure assessment (qSOFA) scores, systemic inflammatory response syndrome (SIRS) scores, coexisting comorbidities, Charlson comorbidity index (CCI) values [19], and many laboratory findings at admission were recorded. In addition, the presence of concomitant pancreatitis or cholecystitis, the etiology of AC, time from admission to biliary drainage, method of biliary drainage, complications related to biliary drainage, length of hospital stay, frequency of ICU admission, the presence of bacteremia, and the type of bacteremia were recorded.

### 2.3. Statistical Analysis

IBM SPSS v. 26.0 software for Windows (IBM Corp., Armonk, NY, USA) was used for the statistical analyses. The normality of the data distribution was checked with the Kolmogorov–Smirnov test. Normally distributed data were shown as mean ± standard deviation and non-normally distributed data were shown as median (interquartile range) values. A Student’s *t*-test or the Mann–Whitney U test was used for continuous variables, whereas the chi-square test and Fisher’s exact test were used to compare categorical variables. A univariate logistic regression analysis was performed with parameters that were likely to be associated with in-hospital mortality based on the results of previous studies. Parameters found to be associated with mortality at the *p* < 0.1 level in the univariate analysis were included in a forward stepwise multivariate logistic regression analysis to determine the independent predictors of in-hospital mortality. The odds ratios (ORs) of the parameters were calculated with 95% confidence intervals (CIs). The area under the curve (AUC) values of the independent predictors of in-hospital mortality were calculated using a receiver operating characteristic (ROC) curve analysis. The optimal cut-off values of the independent predictors were determined based on Youden’s method using the ROC curve. At their optimal cut-off values, the sensitivity, specificity, positive predictive value (PPV) and negative predictive value (NPV) of the predictive parameters were also calculated, and a risk analysis was performed. A *p* value of <0.05 was considered statistically significant in all analyses.

## 3. Results

### 3.1. Comparative Baseline Characteristics and Clinical Outcomes

Of the 250 patients, 87 (34.8%) were in the octogenarian group and 163 (65.2%) were in the non-octogenarian group. The median age of the entire population was 76 (69–82) years. Of the patients, 125 (50%) were female and 125 (50%) were male. The proportion of female patients was higher in the octogenarian group (44.8% vs. 59.8%, *p* = 0.024). Abdominal pain, jaundice, and fever were present as initial complaints in 96.4%, 31.6%, and 16.4% of the patients, respectively. Charcot’s triad was present in only 17 (6.8%) patients. A total of seven (2.8%) patients had Reynolds’ pentad, with three (3.4%) patients in the octogenarian group and four (2.4%) patients in the non-octogenarian group. The octogenarian group had a lower oxygen saturation (*p* = 0.042). There was no significant difference between the two groups in terms of other vital parameters. Disturbance of consciousness (GCS < 15) was higher in the octogenarian group (*p* = 0.042). The etiology of cholangitis was biliary stones in 194 (77.6%) patients, malignancy in 51 (20.4%), and benign biliary stricture in five (2%). The rate of severe disease (grade 3) at admission was significantly higher in the octogenarian group compared to the non-octogenarian group (26.4% vs. 16.6%, respectively, *p* < 0.001). The remaining clinical and demographic characteristics are summarized in Table 1.

The median urea level was higher in the octogenarian group (*p* < 0.001). The median albumin level was lower in the octogenarian group (*p* = 0.001). The median serum calcium and gamma-glutamyl transferase levels were lower in the octogenarian group (*p* = 0.013 and *p* = 0.001, respectively). The remaining laboratory values examined showed no significant differences between the two groups. The laboratory results are shown in Table 2.

**Figure 1:**
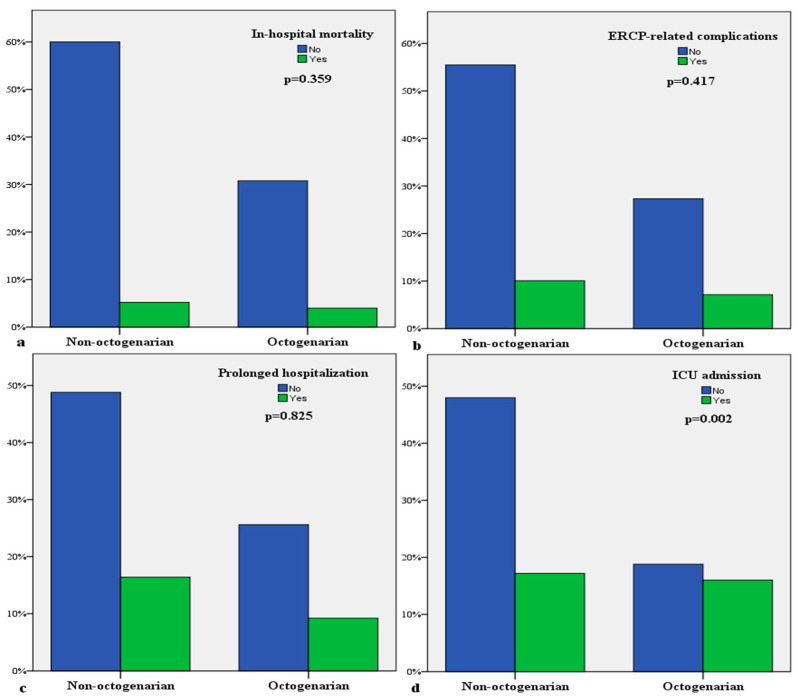
Comparative bar diagrams of clinical outcomes: (**a**) In-hospital mortality; (**b**) ERCP-related complications; (**c**) prolonged hospitalization; (**d**) ICU admission.

A total of 180 (72%) patients underwent biliary drainage, which was performed using ERCP in 155 (86%) and percutaneous transhepatic cholangiography (PTC) in 25 (14%). There was no significant difference between the two groups in terms of biliary drainage procedures (*p* = 0.485). The median length of hospital stay was 11 days, indicating no significant difference between the groups (*p* = 0.587). ICU admission was required by 46% of the patients in the octogenarian group and 26.4% of those in the non-octogenarian group (*p* = 0.002). No significant difference was observed in complications related to ERCP between the two groups (*p* = 0.417). Although the in-hospital mortality rate was higher in the octogenarian group, the difference was not statistically significant (*p* = 0.359) (Figure 1). The comparison of the remaining clinical outcomes is given in Table 3.

### 3.2. Factors Predicting In-Hospital Mortality in the Entire Population

Univariate and multivariate regression analyses were performed to investigate factors predicting in-hospital mortality in the whole population. While many parameters were associated with in-hospital mortality in the univariate analysis, only malignant etiology (OR: 5.68, 95% CI: 2.11–15.3, *p* < 0.001), hypoalbuminemia (OR: 0.18, 95% CI: 0.07–0.45, *p* < 0.001), and qSOFA score (OR: 6.5, 95% CI: 1.7–24.5, *p* = 0.006) were identified as independent risk factors in the multivariate analysis. Table 4 shows the results of the univariate and multivariate regression analyses for in-hospital mortality.

**Figure 2:**
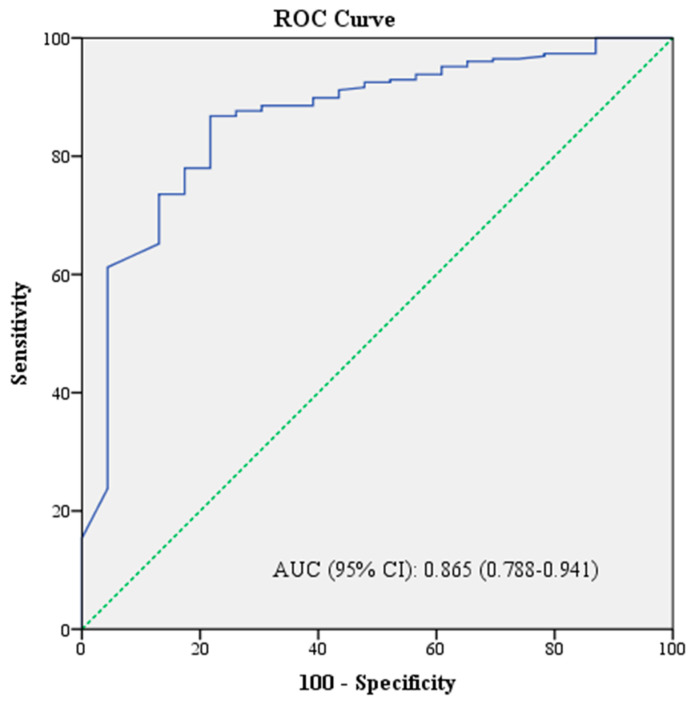
Predictive performance of the combination of malignant etiology, qSOFA score, and albumin for in-hospital mortality.

Using the ROC curve, the optimal cut-off value for albumin was determined to be 3.5 g/dL according to Youden’s method. Patients with an albumin value below 3.5 g/dL had a 3.8-fold increased risk of death (OR: 3.8, 95% CI: 1.6–9). In addition, the risk of death was 8-fold higher in patients with malignant etiology (OR: 8, 95% CI: 3.2–19.8) and 7.7-fold higher in those with a qSOFA score of ≥2 (OR: 7.7, 95% CI: 2.5–23.6) (Table 5). ROC analysis was used to assess the predictive performance of the combination of the parameters in the multivariate analysis for mortality. Accordingly, the AUC value of the combination of malignant etiology, hypoalbuminemia, and qSOFA score ≥ 2 was 0.865 (95% CI: 0.788–0.941) for predicting in-hospital mortality (Figure 2).

## 4. Discussion

In this prospective study, where we thoroughly compared the course of AC in octogenarian (over 80 years) and non-octogenarian (65–79 years) patients, we found no significant difference between the two groups in terms of in-hospital mortality and complications related to ERCP. Additionally, in the elderly population (over 65 years), we identified hypoalbuminemia, malignant etiology, and the qSOFA score as independent predictors of in-hospital mortality.

While Charcot’s triad was commonly used in the past for the diagnosis of AC, it has been replaced by the Tokyo Guidelines due to the low diagnostic accuracy of Charcot’s criteria [17]. Elderly patients in particular may not exhibit a clear fever response and other symptoms of AC, which may lead to a lower number of patients meeting Charcot’s criteria and increase the risk of overlooked AC diagnoses. Supporting this, in the current study, only 6.8% of the patients had Charcot’s triad. Recent studies also show similarly low rates of Charcot’s triad in elderly patients [15,20]. Indeed, in our study, the diagnosis and severity classification of AC were made according to the updated Tokyo Guidelines (TG18).

The increase in comorbidities with age and delays in diagnosis due to the presence of vague symptoms suggest that the clinical prognosis of AC may be worse in elderly patients. Indeed, several studies have observed that advanced age worsens prognosis [16,21,22,23]. However, there are also studies showing the opposite. In a study conducted by Park et al., no difference was found in mortality between the octogenarian and non-octogenarian groups [15]. In another study using propensity score matching analysis, no difference in mortality was found between the octogenarian and non-octogenarian groups [24]. In our study, although the rates of inotrope requirements and in-hospital mortality were higher in the octogenarian group, no statistically significant difference was found between the two groups. One reason for the lack of significant differences may be the study’s limited power; larger studies might reveal significant differences between the groups. The varying results in the literature regarding AC mortality in the elderly suggest that outcomes may be influenced by several factors, such as the physical facilities of the hospital, the timing of interventional procedures, and the experience of the teams performing them. Although age is considered a poor prognostic factor in the TG18, the impact of advanced age on prognosis continues to be a subject of ongoing debate.

The specific complications of ERCP, such as acute pancreatitis, bleeding, and perforation, are seen as a significant concern, particularly in elderly patients. A large meta-analysis investigating ERCP-related complications found bleeding, cardiopulmonary adverse events, and mortality to be more common in patients over 80 years of age [25]. However, the authors did not observe any differences when comparing patient groups over and under the age of 65 [25]. In contrast, our study did not demonstrate a significant difference in complications between the octogenarian and non-octogenarian groups. This may be due to our comparison of octogenarians with a non-octogenarian but elderly patient group aged 65–79 years. Another study with a median age of 67 in the non-octogenarian group supports our findings, also reporting no increase in complications in octogenarian patients, and interestingly noted that post-ERCP pancreatitis was less common in the octogenarian group [13]. Furthermore, other studies suggest that, with aging, pancreatic atrophy and fibrosis increase, potentially leading to a lower risk of post-ERCP pancreatitis in the elderly population [14]. As demonstrated by Ramai et al., frailty, in addition to age, is also a crucial factor in the development of complications [26]. The presence of varying data in the literature regarding ERCP-related complications suggests that factors such as patients’ comorbidities, the timing of the procedure, disease severity, medications used, and the clinician’s experience may all impact the rate of complications, independent of age.

In patients diagnosed with AC, determining the severity of the disease at the time of diagnosis and triaging the patient accordingly is important. For this purpose, we investigated the factors predicting in-hospital mortality by evaluating admission clinical and laboratory parameters in the entire population. There are limited data in the literature investigating factors associated with mortality, particularly in elderly patients. A study including patients with cholangitis and cholecystitis found that advanced age, cancer, the CCI score, bacteremia, and disease severity were predictive of mortality [27]. Similarly, in a study by Park et al., the univariate analysis revealed that disease severity and success of biliary drainage were predictors of mortality, although the authors did not perform a multivariate analysis [15]. Another study, evaluating critically ill patients with severe cholangitis, identified malnutrition and the SOFA score as factors associated with six-month mortality [28]. While several other studies in the literature have examined factors related to mortality, they have all been retrospective in nature [20,29]. In our prospective study, malignant etiology, hypoalbuminemia, and the qSOFA score were identified as independent risk factors for in-hospital mortality. Furthermore, the combination of these three parameters was shown to predict mortality with a high AUC value of 0.865 in the ROC analysis.

It is important to note that in elderly patients diagnosed with AC, hypoalbuminemia, malignant etiology, or a qSOFA score of ≥2 at the time of admission may indicate a poor prognosis. Although clinicians typically base their decision for biliary drainage on the TG18, our study results suggest that these three parameters predict AC mortality more accurately than TG18. Therefore, recognizing the potential for poor outcomes in elderly patients with these parameters suggests that close monitoring and earlier biliary drainage may be a prudent approach. However, no additional analysis on the timing of biliary drainage was conducted in our study, and further research is needed for this purpose. Moreover, hypoalbuminemia is listed as a grade 2 criterion in the TG18, but, considering that hypoalbuminemia is a strong predictor of mortality, it may be more reasonable to classify it as a grade 3 criterion. In this context, our study’s findings may guide future updates to the guidelines. Clinicians may have reservations about performing ERCP in very elderly patients, but the lack of increased ERCP-related complications in the octogenarian group in our study demonstrates that clinicians can safely perform ERCP in this population when necessary.

Our study has several limitations, the primary one being its single-center design. However, as our hospital is a tertiary care center with the highest number of AC cases in Turkey, serving a diverse patient population referred from various hospitals, this limitation may be mitigated. Additionally, the study’s observational design poses a limitation. Different treatment procedures may have been applied due to the inherent age difference between the two groups. Clinical decisions, such as ICU admission and the timing of ERCP procedures, could have been influenced by patient age, potentially leading to selection bias. Therefore, a randomized controlled trial design would help eliminate selection bias and confounding factors, providing more definitive results. Moreover, the inclusion of a heterogeneous patient population, including those with spontaneous biliary drainage or a diagnosis of malignancy, introduces potential confounding variables, such as malignancy stage and the presence of metastasis. Nevertheless, the inclusion of all patient groups enhances the study’s value by providing real-world data. Furthermore, our study did not assess functional, nutritional, mental, or frailty parameters. A more comprehensive geriatric assessment could have better identified mortality predictors in the octogenarian group. Finally, although our hospital is a highly experienced center, the involvement of multiple physicians in performing interventional procedures like ERCP could also have been a confounding factor for certain outcomes, such as complications.

In conclusion, this study provides a detailed comparison between octogenarian and non-octogenarian patient groups across various parameters. Being over 80 years old is not an indicator of poor outcomes, and ERCP can be safely performed on these patients. However, elderly patients with hypoalbuminemia, malignant etiology, or a qSOFA score ≥ 2 should be closely monitored, regardless of age. Further large-scale, multicenter studies are needed to better understand the course of AC in octogenarian patients and to confirm our findings.

## Figures and Tables

**Table 1:** Comparison of clinical and demographic characteristics.

Parameter	Overall n = 250	Non-Octogenarian n = 163	Octogenarian n = 87	*p*
Age, years	76 (69–82)	73 (68–76)	84 (82–88)	<0.001
Female gender	125 (50%)	73 (44.8%)	52 (59.8%)	**0.024**
Etiology				0.093
Biliary stones	194 (77.6%)	120 (73.6%)	74 (85%)	
Malignant biliary obstruction	51 (20.4%)	38 (23.3%)	13 (14.9%)	
Benign biliary stricture	5 (2%)	5 (3.1%)	0	
Main complaint at admission				
Abdominal pain	241 (96.4%)	157 (96.3%)	84 (96.6%)	0.925
Jaundice	79 (31.6%)	51 (31.3%)	28 (32.2%)	0.885
Fever	41 (16.4%)	27 (16.6%)	14 (16.1%)	0.923
Charcot’s triad	17 (6.8%)	12 (7.4%)	5 (5.7%)	0.629
Vital signs				
Mean arterial pressure	89 ± 14	90 ± 14	87 ± 14	0.067
Heart rate per minute	85 (78–92)	85 (78–91)	85 (80–95)	0.389
Respiratory rate per minute	16 (15–18)	16 (14–18)	16 (15–18)	0.150
Oxygen saturation, %	94 (92–96)	95 (93–96)	93 (91–96)	**0.010**
Disturbance of consciousness (GCS score < 15)	29 (11.6%)	14 (8.6%)	15 (17.2%)	**0.042**
TG18 severity grading				**<0.001**
Grade 1 (mild)	112 (44.8%)	89 (54.6%)	23 (26.4%)	
Grade 2 (moderate)	88 (35.2%)	47 (28.8%)	41 (47.1%)	
Grade 3 (severe)	50 (20%)	27 (16.6%)	23 (26.4%)	
qSOFA score, ≥2	16 (6.4%)	8 (4.9%)	8 (9.2%)	0.187
SIRS score, ≥2	77 (30.8%)	46 (28.2%)	31 (35.6%)	0.227
Concomitant acute pancreatitis	68 (27.2%)	45 (27.6%)	23 (26.4%)	0.843
Concomitant acute cholecystitis	63 (25.2%)	40 (24.5%)	23 (26.4%)	0.742
History of cholecystectomy	41 (16.4%)	26 (16%)	15 (17.2%)	0.707
History of acute cholangitis	50 (20%)	32 (19.6%)	18 (20.7%)	0.744
Charlson comorbidity index	2 (1–4)	2 (1–4)	2 (1–4)	0.764

GCS, Glasgow Coma Scale; SIRS, systemic inflammatory response syndrome; qSOFA, quick sequential organ failure assessment; TG18, Tokyo Guidelines 2018.

**Table 2:** Comparison of laboratory data between the octogenarian and non-octogenarian groups.

Parameter	Overall n = 250	Non-Octogenarian n = 163	Octogenarian n = 87	*p*
White blood cell count, 10^9^/L	10.7 (7.7–14.3)	10.8 (7.9–14.3)	10.3 (7.3–14.5)	0.552
Neutrophil count, 10^9^/L	9 (5.9–12.7)	9.2 (6–12.7)	8.3 (5.5–12.9)	0.633
Hemoglobin, g/dL	12.8 ± 1.8	13 ± 1.8	12.5 ± 1.75	0.052
Platelet count, 10^9^/L	226 (180–289)	225 (186–289)	228 (172–296)	0.941
ALT, U/L	178 (101–278)	186 (104–286)	150 (91–251)	0.147
AST, U/L	177 (99–281)	165 (99–272)	194 (96–329)	0.253
ALP, U/L	267 (170–452)	269 (172–460)	262 (159–452)	0.491
GGT, U/L	357 (212–568)	379 (240–665)	270 (194–475)	**0.001**
Total bilirubin, mg/dL	3.7 (2.2–6.4)	3.8 (2.2–6.7)	3.2 (1.9–6.4)	0.210
INR	1.15 (1.09–1.27)	1.16 (1.1–1.26)	1.12 (1.02–1.3)	0.225
Amylase, U/L	62 (39–391)	62 (35–385)	69 (41–423)	0.555
Lipase, U/L	45 (29–495)	41 (30–417)	51 (28–667)	0.945
Urea, mg/dL	42 (34–56)	41 (31–54)	47 (39–64)	**<0.001**
Creatinine, mg/dL	0.9 (0.7–1.2)	0.9 (0.7–1.1)	1 (0.8–1.2)	0.070
Albumin, g/dL	3.8 (3.5–4.1)	3.9 (3.5–4.2)	3.7 (3.4–3.9)	**0.001**
C-reactive protein, mg/L	65 (25–122)	65 (25–122)	61 (25–135)	0.902
Procalcitonin, µg/L	0.81 (0.24–5.93)	0.64 (0.21–5.5)	1.43 (0.33–6.92)	0.051
Sodium, mEq/L	138 (135–140)	139 (136–140)	138 (135–140)	0.460
Potassium, mEq/L	4.1 (3.8–4.4)	4.1 (3.8–4.4)	4.1 (3.8–4.5)	0.902
Calcium, mg/dL	8.7 (8.3–9.1)	8.8 (8.4–9.1)	8.6 (8.2–9)	**0.013**
Lactate, mmol/L	2.03 (1.55–2.80)	2.06 (1.5–2.74)	1.99 (1.62–2.97)	0.729

ALT, alanine aminotransferase; AST, aspartate aminotransferase; ALP, alkaline phosphatase; GGT, gamma-glutamyl transferase; INR, international normalized ratio.

**Table 3:** Comparative clinical outcomes of the patients.

Parameter	Overall n = 250	Non-Octogenarian n = 163	Octogenarian n = 87	*p*
Biliary drainage	180 (72%)	115 (70.6%)	65 (74.7%)	0.485
ERCP	155 (62%)	97 (59.5%)	58 (66.7%)	
PTC	25 (10%)	18 (11%)	7 (8%)	
None	70 (28%)	48 (29.4%)	22 (25.3%)	
Time to drainage, hour	120 (36–192)	120 (42–192)	96 (35–173)	0.526
Length of hospital stay, day	11 (7–17)	10 (7–17)	12 (8–17)	0.587
Prolonged hospitalization, >75th percentile	64 (25.6%)	41 (25.2%)	23 (26.4%)	0.825
ICU admission	83 (33.2%)	43 (26.4%)	40 (46%)	**0.002**
Length of ICU stay, day	7 (4–13)	7 (4–14)	7 (4–13)	0.964
ERCP-related complications	38 (21.1%)	21 (18.3%)	17 (26.2%)	0.417
Pancreatitis	33 (18.3%)	19 (16.5%)	14 (21.5%)	
Bleeding	4 (2.2%)	2 (1.74%)	2 (3.08%)	
Perforation	1 (0.55%)	0	1 (1.54%)	
Anesthesia-related complication	3 (1.66%)	3 (2.6%)	0	
Inotrope requirement	33 (13.2%)	19 (11.7%)	14 (16.1%)	0.324
In-hospital mortality	23 (9.2%)	13 (8%)	10 (11.5%)	0.359
Bacteremia	49 (19.6%)	28 (17.2%)	21 (24.1%)	0.187
Gram-negative	32 (65.3%)	18 (64.3%)	14 (66.7%)	
Gram-positive	16 (34.7%)	10 (35.7%)	6 (28.6%)	

ERCP, endoscopic retrograde cholangiopancreatography; PTC, percutaneous transhepatic cholangiography; ICU, intensive care unit.

**Table 4:** Parameters predicting in-hospital mortality.

Parameter	Univariate Analysis		Multivariate Analysis	
	OR (95% CI)	*p*	OR (95% CI)	*p*
Age	1.04 (0.98–1.10)	0.166		
Female gender	1.99 (0.81–4.89)	0.131		
Malignant etiology	7.99 (3.22–19.81)	**<0.001**	5.68 (2.11–15.3)	**<0.001**
Systolic blood pressure	0.97 (0.95–0.99)	0.003		
Hemoglobin	0.77 (0.61–0.98)	0.032		
Platelet count	1 (0.99–1.01)	0.134		
Total bilirubin	1.11 (1.04–1.19)	0.002		
INR	1.71 (0.53–5.53)	0.368		
Creatinine	0.66 (0.26–1.66)	0.375		
Albumin	0.14 (0.06–0.32)	**<0.001**	0.18 (0.07–0.45)	**<0.001**
C-reactive protein	1 (0.99–1.01)	0.903		
Procalcitonin	1 (0.99–1.01)	0.861		
TG18 severity grading	2.34 (0.94–5.90)	0.069		
Lactate	1.31 (0.94–1.85)	0.115		
Bacteremia	8.533 (3.43–21.2)	<0.001		
Concomitant pancreatitis	0.723 (0.26–2.03)	0.538		
Concomitant cholecystitis	0.599 (0.2–1.83)	0.370		
Charlson comorbidity index	2.06 (1.17–3.8)	0.021		
SIRS score ≥ 2	2.72 (1.14–6.47)	0.024		
qSOFA score ≥ 2	7.66 (2.48–23.61)	**<0.001**	6.5 (1.7–24.5)	**0.006**
GCS score < 15	8.42 (3.26–21.75)	<0.001		

OR, odds ratio; CI, confidence interval; SIRS, systemic inflammatory response syndrome; qSOFA, quick sequential organ failure assessment; INR, international normalized ratio; GCS, Glasgow Coma Scale.

**Table 5:** Predictive abilities of independent risk factors for in-hospital mortality at the optimal cut-off values.

	Cut-Off Value	Number (%) of Patients *	OR (95% CI)	Sens %	Spec %	PPV %	NPV %
Albumin	<3.5 mg/dL	63 (25.2)	3.8 (1.6–9)	52.2	77.5	19	94.1
Malignant etiology	-	51 (20.4)	8 (3.2–19.8)	60.9	83.7	27.5	95.5
qSOFA score ≥ 2	-	16 (6.4)	7.7 (2.5–23.6)	26.1	95.6	37.5	92.7

CI, confidence interval; Sens, sensitivity; Spec, specificity; PPV, positive predictive value; NPV, negative predictive value; OR, odds ratio. * Number (%) of patients with values above or below the given cut-off values.

## Data Availability

Data from the study are not openly available to other researchers due to protected patient information. Further inquiries can be directed to the corresponding author.
